# 5-Hy­droxy-3,4′,6,7-tetra­meth­oxy­flavone

**DOI:** 10.1107/S160053681103827X

**Published:** 2011-09-30

**Authors:** Hua-Wei Geng, Guo-Cai Wang, Guo-Qiang Li, Ren-Wang Jiang, Yao-Lan Li

**Affiliations:** a Guangdong Province Key Laboratory of Pharmacodynamic Constituents of Traditional Chinese Medicine and New Drugs Research, Institute of Traditional Chinese Medicine and Natural Products, College of Pharmacy, Jinan University, Guangzhou 510632, People’s Republic of China

## Abstract

The title compound, C_19_H_18_O_7_ [systematic name 5-hy­droxy-3,6,7-tri­meth­­oxy-2-(4-meth­oxy­phen­yl)-4*H*-1-benzopyran-4-one], is a flavonoid which was isolated from the traditional Chinese medicine Laggera alata. The benzene ring of the benzopyran­one unit forms dihedral angles of 1.72 (3) and 37.39 (5)° with the pyran ring and the substituent benzene ring, respectively. The mol­ecular conformation is stabilized by an intra­molecular phenol O—H⋯O_ketone_ hydrogen bond.

## Related literature

For general background to the synthesis and isolation of the title compound, see: Goldsworthy & Robert (1936 ▶); Sy & Brown (1998 ▶); Yang *et al.* (2007 ▶); Masateru *et al.* (2009 ▶). For its anti-hepatotoxic activity, see: Chhaya & Mishra (2007 ▶).
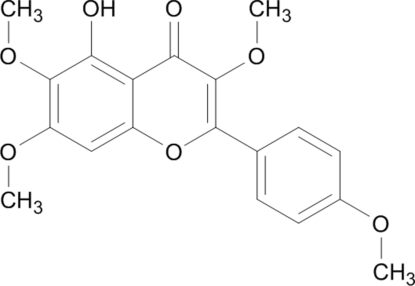


## Experimental

### 

#### Crystal data

C_19_H_18_O_7_
*M* *_r_* = 358.33
Monoclinic, 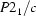

*a* = 16.6029 (3) Å
*b* = 7.40255 (12) Å
*c* = 14.8666 (3) Å
β = 110.487 (2)°
*V* = 1711.60 (6) Å^3^
*Z* = 4
Cu *K*α radiation
μ = 0.90 mm^−1^
*T* = 295 K
0.26 × 0.21 × 0.18 mm

#### Data collection

Oxford Diffraction Xcalibur Sapphire3 Gemini Ultra CCD diffractometer
Absorption correction: multi-scan (*CrysAlis PRO*; Agilent, 2011 ▶) *T*_min_ = 0.596, *T*_max_ = 1.000
5568 measured reflections
2681 independent reflections
2380 reflections with *I* > 2σ(*I*)
*R*_int_ = 0.016

#### Refinement

*R*[*F* ^2^ > 2σ(*F* ^2^)] = 0.035
*wR*(*F* ^2^) = 0.097
*S* = 1.03
2681 reflections
241 parameters
H-atom parameters constrained
Δρ_max_ = 0.14 e Å^−3^
Δρ_min_ = −0.13 e Å^−3^

### 

Data collection: *CrysAlis PRO* (Agilent, 2011 ▶); cell refinement: *CrysAlis PRO*; data reduction: *CrysAlis PRO*; program(s) used to solve structure: *SHELXS97* (Sheldrick, 2008 ▶); program(s) used to refine structure: *SHELXL97* (Sheldrick, 2008 ▶); molecular graphics: *OLEX2* (Dolomanov *et al.*, 2009 ▶); software used to prepare material for publication: *OLEX2*.

## Supplementary Material

Crystal structure: contains datablock(s) I, global. DOI: 10.1107/S160053681103827X/zs2143sup1.cif

Structure factors: contains datablock(s) I. DOI: 10.1107/S160053681103827X/zs2143Isup2.hkl

Supplementary material file. DOI: 10.1107/S160053681103827X/zs2143Isup3.cml

Additional supplementary materials:  crystallographic information; 3D view; checkCIF report

## Figures and Tables

**Table 1:** Hydrogen-bond geometry (Å, °)

*D*—H⋯*A*	*D*—H	H⋯*A*	*D*⋯*A*	*D*—H⋯*A*
O4—H4⋯O5	0.82	1.89	2.6157 (16)	147

**Fig. 1.**
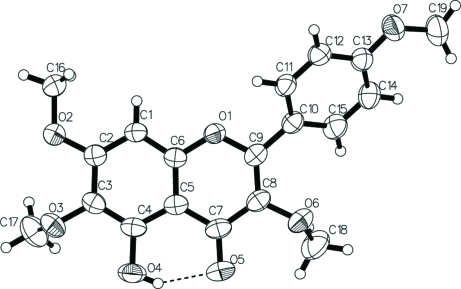
The molecular structure of the title compound showing 50% probability displacement ellipsoids and the atom-numbering scheme.

## supplementary crystallographic information

### Comment

The title compound, the flavonoid C_19_H_18_O_7_ [systematic name 5-hydroxy-3,6,7-trimethoxy-2-(4-methoxyphenyl)-4*H*-1-benzopyran-4-one], (Fig. 1) was originally sythesised from trimethoxyacetophenone (Goldsworthy & Robert, 1936). It was also isolated from Artemisia annua (Sy & Brown, 1998), Laggera pterodonta (Yang *et al.*, 2007) and Aites agnus-castus (Masateru *et al.*, 2009). The flavonoid was also proved to possess significant anti-hepatotoxic activity (Chhaya *et al.*, 2007). The present compound was isolated from the traditional Chinese medicine Laggera alata. In the crystal structure, the dihedral angle between the plane of the benzene ring *A* and the pyran plane *C* is 1.72 (3)°, while that between the benzene ring *A* and the phenyl ring *B* is 37.39 (5)°. The molecular conformation is stabilized by an intramolecular phenol O—H···O_ketone_ hydrogen-bonding interaction (Table 1).

### Experimental

The title compound was isolated from the herbs of the traditional Chinese medicine Laggera alata. The herbs of Laggera alata (5 kg) was extracted with 95% ethanol at room temperature and the extracted solution was concentrated by rotary evaporator. The crude extract was suspended in distilled water and partitioned with petroleum ether, ethyl acetate and *n*-butanol. The title compound (50 mg) was isolated from the petroleum ether fraction using silica gel column chromatography and crystals were obtained after slow evaporation of an ethyl acetate solution at room temperature.

### Refinement

The C-bound H atoms were positioned geometrically and were included in the refinement in the riding-model approximation, with C—H = 0.96 Å (CH_3_) ,0.93 Å (aryl H) and O—H = 0.82 Å and with *U*_iso_(H) = 1.2*U*_eq_(C) (aryl H) and = 1.5*U*_eq_[C(methyl) and O].

### Crystal data

**Table:**

C_19_H_18_O_7_	*F*(000) = 752
*M**_r_* = 358.33	*D*_x_ = 1.391 Mg m^−^^3^
Monoclinic, *P*2_1_/*c*	Cu *K*α radiation, λ = 1.5418 Å
*a* = 16.6029 (3) Å	Cell parameters from 3031 reflections
*b* = 7.40255 (12) Å	θ = 3.2–62.6°
*c* = 14.8666 (3) Å	µ = 0.90 mm^−^^1^
β = 110.487 (2)°	*T* = 295 K
*V* = 1711.60 (6) Å^3^	Block, colourless
*Z* = 4	0.26 × 0.21 × 0.18 mm

### Data collection

**Table:**

Oxford Diffraction Xcalibur Sapphire3 Gemini Ultra CCD diffractometer	2681 independent reflections
Radiation source: Enhance Ultra (Cu) X-ray Source	2380 reflections with *I* > 2σ(*I*)
mirror	*R*_int_ = 0.016
Detector resolution: 16.0288 pixels mm^-1^	θ_max_ = 62.7°, θ_min_ = 5.7°
ω scans	*h* = −19→18
Absorption correction: multi-scan (*CrysAlis PRO*; Agilent, 2011)	*k* = −8→8
*T*_min_ = 0.596, *T*_max_ = 1.000	*l* = −12→17
5568 measured reflections	

### Refinement

**Table:**

Refinement on *F*^2^	Primary atom site location: structure-invariant direct methods
Least-squares matrix: full	Secondary atom site location: difference Fourier map
*R*[*F*^2^ > 2σ(*F*^2^)] = 0.035	Hydrogen site location: inferred from neighbouring sites
*wR*(*F*^2^) = 0.097	H-atom parameters constrained
*S* = 1.03	*w* = 1/[σ^2^(*F*_o_^2^) + (0.0545*P*)^2^ + 0.2624*P*] where *P* = (*F*_o_^2^ + 2*F*_c_^2^)/3
2681 reflections	(Δ/σ)_max_ = 0.001
241 parameters	Δρ_max_ = 0.14 e Å^−^^3^
0 restraints	Δρ_min_ = −0.13 e Å^−^^3^

### Special details

**Table:**

Geometry. All esds (except the esd in the dihedral angle between two l.s. planes) are estimated using the full covariance matrix. The cell esds are taken into account individually in the estimation of esds in distances, angles and torsion angles; correlations between esds in cell parameters are only used when they are defined by crystal symmetry. An approximate (isotropic) treatment of cell esds is used for estimating esds involving l.s. planes.
Refinement. Refinement of *F*^2^ against ALL reflections. The weighted *R*-factor *wR* and goodness of fit *S* are based on *F*^2^, conventional *R*-factors *R* are based on *F*, with *F* set to zero for negative *F*^2^. The threshold expression of *F*^2^ > σ(*F*^2^) is used only for calculating *R*-factors(gt) *etc*. and is not relevant to the choice of reflections for refinement. *R*-factors based on *F*^2^ are statistically about twice as large as those based on *F*, and *R*- factors based on ALL data will be even larger.

### Fractional atomic coordinates and isotropic or equivalent isotropic displacement parameters (Å^2^)

**Table:**

	*x*	*y*	*z*	*U*_iso_*/*U*_eq_	
C1	0.48173 (9)	0.62713 (18)	0.35189 (9)	0.0426 (3)	
H1	0.4827	0.6247	0.4148	0.051*	
C2	0.55652 (9)	0.65137 (18)	0.33208 (9)	0.0426 (3)	
C3	0.55508 (9)	0.65911 (18)	0.23692 (10)	0.0437 (3)	
C4	0.47856 (10)	0.63752 (18)	0.16217 (9)	0.0452 (4)	
C5	0.40106 (9)	0.60851 (18)	0.18019 (9)	0.0425 (3)	
C6	0.40584 (9)	0.60677 (17)	0.27555 (9)	0.0400 (3)	
C7	0.31987 (10)	0.58282 (19)	0.10407 (10)	0.0470 (4)	
C8	0.24726 (9)	0.55273 (19)	0.13451 (10)	0.0463 (4)	
C9	0.25552 (9)	0.55542 (18)	0.22864 (9)	0.0426 (3)	
C10	0.18754 (9)	0.54065 (19)	0.27030 (9)	0.0438 (3)	
C11	0.19372 (9)	0.6426 (2)	0.35134 (10)	0.0471 (4)	
H11	0.2413	0.7167	0.3786	0.057*	
C12	0.13104 (9)	0.6358 (2)	0.39175 (10)	0.0512 (4)	
H12	0.1363	0.7058	0.4455	0.061*	
C13	0.05974 (9)	0.5249 (2)	0.35272 (10)	0.0502 (4)	
C14	0.05317 (10)	0.4198 (2)	0.27362 (12)	0.0581 (4)	
H14	0.0063	0.3432	0.2478	0.070*	
C15	0.11659 (10)	0.4286 (2)	0.23276 (11)	0.0553 (4)	
H15	0.1114	0.3581	0.1792	0.066*	
C16	0.64117 (10)	0.6460 (3)	0.49839 (11)	0.0672 (5)	
H16A	0.6228	0.5260	0.5065	0.101*	
H16B	0.6050	0.7324	0.5140	0.101*	
H16C	0.6996	0.6630	0.5402	0.101*	
C17	0.67982 (12)	0.5470 (3)	0.21626 (15)	0.0744 (5)	
H17A	0.6467	0.4644	0.1676	0.112*	
H17B	0.6983	0.4879	0.2776	0.112*	
H17C	0.7292	0.5860	0.2020	0.112*	
C18	0.15121 (13)	0.3816 (3)	0.00626 (14)	0.0818 (6)	
H18A	0.1759	0.4002	−0.0425	0.123*	
H18B	0.0905	0.3617	−0.0234	0.123*	
H18C	0.1773	0.2782	0.0442	0.123*	
C19	−0.07302 (12)	0.4180 (3)	0.36159 (15)	0.0807 (6)	
H19A	−0.1054	0.4487	0.2961	0.121*	
H19B	−0.1084	0.4341	0.4001	0.121*	
H19C	−0.0549	0.2942	0.3650	0.121*	
O1	0.33340 (6)	0.58608 (13)	0.29847 (6)	0.0426 (3)	
O2	0.63532 (6)	0.67071 (15)	0.40059 (7)	0.0525 (3)	
O3	0.62890 (7)	0.69783 (14)	0.21861 (7)	0.0529 (3)	
O4	0.47794 (8)	0.64402 (17)	0.07088 (7)	0.0620 (3)	
H4	0.4288	0.6281	0.0334	0.093*	
O5	0.31225 (8)	0.58768 (17)	0.01722 (7)	0.0616 (3)	
O6	0.16597 (7)	0.53866 (16)	0.06686 (7)	0.0587 (3)	
O7	0.00047 (7)	0.53200 (18)	0.39654 (8)	0.0674 (4)	

### Atomic displacement parameters (Å^2^)

**Table:**

	*U*^11^	*U*^22^	*U*^33^	*U*^12^	*U*^13^	*U*^23^
C1	0.0497 (8)	0.0430 (8)	0.0356 (7)	−0.0008 (6)	0.0155 (6)	0.0017 (6)
C2	0.0492 (8)	0.0354 (7)	0.0431 (7)	−0.0009 (6)	0.0160 (6)	0.0014 (6)
C3	0.0547 (8)	0.0332 (7)	0.0489 (8)	−0.0023 (6)	0.0253 (7)	0.0009 (6)
C4	0.0654 (9)	0.0362 (7)	0.0389 (7)	−0.0008 (6)	0.0243 (7)	0.0009 (6)
C5	0.0552 (8)	0.0343 (7)	0.0380 (7)	0.0016 (6)	0.0165 (6)	0.0008 (5)
C6	0.0486 (8)	0.0334 (7)	0.0396 (7)	0.0022 (6)	0.0175 (6)	0.0026 (5)
C7	0.0626 (9)	0.0394 (7)	0.0359 (7)	0.0056 (7)	0.0132 (6)	0.0033 (6)
C8	0.0513 (8)	0.0415 (8)	0.0394 (7)	0.0053 (6)	0.0074 (6)	0.0014 (6)
C9	0.0469 (8)	0.0343 (7)	0.0410 (7)	0.0030 (6)	0.0084 (6)	−0.0001 (6)
C10	0.0448 (7)	0.0396 (7)	0.0418 (7)	0.0011 (6)	0.0086 (6)	0.0013 (6)
C11	0.0455 (8)	0.0488 (8)	0.0424 (7)	−0.0076 (6)	0.0096 (6)	−0.0027 (6)
C12	0.0533 (8)	0.0552 (9)	0.0432 (7)	−0.0080 (7)	0.0143 (6)	−0.0058 (7)
C13	0.0476 (8)	0.0517 (9)	0.0487 (8)	−0.0052 (7)	0.0139 (6)	0.0020 (7)
C14	0.0525 (9)	0.0538 (9)	0.0633 (9)	−0.0156 (7)	0.0143 (7)	−0.0097 (8)
C15	0.0593 (9)	0.0490 (9)	0.0544 (8)	−0.0080 (7)	0.0159 (7)	−0.0123 (7)
C16	0.0522 (9)	0.0982 (14)	0.0450 (8)	−0.0076 (9)	0.0092 (7)	0.0153 (9)
C17	0.0725 (12)	0.0621 (11)	0.1037 (14)	0.0102 (9)	0.0500 (11)	0.0093 (10)
C18	0.0770 (12)	0.0918 (15)	0.0642 (11)	−0.0132 (11)	0.0088 (9)	−0.0287 (10)
C19	0.0579 (10)	0.0963 (15)	0.0901 (13)	−0.0257 (10)	0.0288 (9)	−0.0111 (12)
O1	0.0438 (5)	0.0456 (5)	0.0367 (5)	−0.0003 (4)	0.0121 (4)	0.0005 (4)
O2	0.0473 (6)	0.0639 (7)	0.0455 (5)	−0.0059 (5)	0.0154 (4)	0.0045 (5)
O3	0.0624 (6)	0.0430 (6)	0.0640 (6)	−0.0042 (5)	0.0357 (5)	0.0008 (5)
O4	0.0791 (8)	0.0728 (8)	0.0406 (5)	−0.0097 (6)	0.0290 (5)	−0.0025 (5)
O5	0.0754 (7)	0.0706 (8)	0.0348 (5)	0.0027 (6)	0.0141 (5)	0.0057 (5)
O6	0.0545 (6)	0.0664 (7)	0.0439 (6)	0.0046 (5)	0.0029 (5)	−0.0018 (5)
O7	0.0587 (7)	0.0816 (9)	0.0676 (7)	−0.0227 (6)	0.0294 (6)	−0.0130 (6)

### Geometric parameters (Å, °)

**Table:**

C1—H1	0.9300	C12—C13	1.390 (2)
C1—C2	1.3845 (19)	C13—C14	1.382 (2)
C1—C6	1.3774 (19)	C13—O7	1.3580 (18)
C2—C3	1.408 (2)	C14—H14	0.9300
C2—O2	1.3552 (17)	C14—C15	1.389 (2)
C3—C4	1.373 (2)	C15—H15	0.9300
C3—O3	1.3755 (17)	C16—H16A	0.9600
C4—C5	1.419 (2)	C16—H16B	0.9600
C4—O4	1.3543 (17)	C16—H16C	0.9600
C5—C6	1.3920 (19)	C16—O2	1.4347 (18)
C5—C7	1.437 (2)	C17—H17A	0.9600
C6—O1	1.3685 (16)	C17—H17B	0.9600
C7—C8	1.446 (2)	C17—H17C	0.9600
C7—O5	1.2531 (17)	C17—O3	1.408 (2)
C8—C9	1.358 (2)	C18—H18A	0.9600
C8—O6	1.3761 (17)	C18—H18B	0.9600
C9—C10	1.469 (2)	C18—H18C	0.9600
C9—O1	1.3644 (16)	C18—O6	1.438 (2)
C10—C11	1.395 (2)	C19—H19A	0.9600
C10—C15	1.388 (2)	C19—H19B	0.9600
C11—H11	0.9300	C19—H19C	0.9600
C11—C12	1.373 (2)	C19—O7	1.424 (2)
C12—H12	0.9300	O4—H4	0.8200
			
C2—C1—H1	121.0	O7—C13—C14	125.10 (14)
C6—C1—H1	121.0	C13—C14—H14	120.0
C6—C1—C2	117.96 (12)	C13—C14—C15	119.95 (14)
C1—C2—C3	121.20 (13)	C15—C14—H14	120.0
O2—C2—C1	123.76 (12)	C10—C15—C14	121.25 (14)
O2—C2—C3	115.04 (12)	C10—C15—H15	119.4
C4—C3—C2	119.61 (13)	C14—C15—H15	119.4
C4—C3—O3	120.00 (12)	H16A—C16—H16B	109.5
O3—C3—C2	120.30 (13)	H16A—C16—H16C	109.5
C3—C4—C5	120.51 (12)	H16B—C16—H16C	109.5
O4—C4—C3	119.16 (13)	O2—C16—H16A	109.5
O4—C4—C5	120.33 (13)	O2—C16—H16B	109.5
C4—C5—C7	122.26 (12)	O2—C16—H16C	109.5
C6—C5—C4	117.47 (13)	H17A—C17—H17B	109.5
C6—C5—C7	120.26 (13)	H17A—C17—H17C	109.5
C1—C6—C5	123.22 (13)	H17B—C17—H17C	109.5
O1—C6—C1	115.93 (11)	O3—C17—H17A	109.5
O1—C6—C5	120.85 (12)	O3—C17—H17B	109.5
C5—C7—C8	115.41 (12)	O3—C17—H17C	109.5
O5—C7—C5	122.41 (14)	H18A—C18—H18B	109.5
O5—C7—C8	122.18 (13)	H18A—C18—H18C	109.5
C9—C8—C7	121.73 (13)	H18B—C18—H18C	109.5
C9—C8—O6	118.29 (14)	O6—C18—H18A	109.5
O6—C8—C7	119.66 (12)	O6—C18—H18B	109.5
C8—C9—C10	128.17 (13)	O6—C18—H18C	109.5
C8—C9—O1	120.90 (13)	H19A—C19—H19B	109.5
O1—C9—C10	110.79 (11)	H19A—C19—H19C	109.5
C11—C10—C9	119.26 (12)	H19B—C19—H19C	109.5
C15—C10—C9	122.96 (13)	O7—C19—H19A	109.5
C15—C10—C11	117.78 (14)	O7—C19—H19B	109.5
C10—C11—H11	119.3	O7—C19—H19C	109.5
C12—C11—C10	121.36 (13)	C9—O1—C6	120.71 (10)
C12—C11—H11	119.3	C2—O2—C16	116.96 (11)
C11—C12—H12	119.9	C3—O3—C17	115.09 (12)
C11—C12—C13	120.29 (14)	C4—O4—H4	109.5
C13—C12—H12	119.9	C8—O6—C18	115.31 (13)
C14—C13—C12	119.34 (14)	C13—O7—C19	118.32 (13)
O7—C13—C12	115.55 (13)		
			
C1—C2—C3—C4	−1.7 (2)	C8—C9—C10—C11	−142.84 (15)
C1—C2—C3—O3	174.72 (12)	C8—C9—C10—C15	37.3 (2)
C1—C2—O2—C16	6.5 (2)	C8—C9—O1—C6	−2.98 (19)
C1—C6—O1—C9	−176.54 (12)	C9—C8—O6—C18	−119.21 (16)
C2—C1—C6—C5	0.3 (2)	C9—C10—C11—C12	178.80 (13)
C2—C1—C6—O1	−178.99 (12)	C9—C10—C15—C14	−179.31 (14)
C2—C3—C4—C5	0.3 (2)	C10—C9—O1—C6	−179.13 (11)
C2—C3—C4—O4	−179.58 (13)	C10—C11—C12—C13	0.5 (2)
C2—C3—O3—C17	88.51 (17)	C11—C10—C15—C14	0.8 (2)
C3—C2—O2—C16	−174.02 (14)	C11—C12—C13—C14	0.9 (2)
C3—C4—C5—C6	1.3 (2)	C11—C12—C13—O7	−178.28 (14)
C3—C4—C5—C7	−179.12 (13)	C12—C13—C14—C15	−1.4 (2)
C4—C3—O3—C17	−95.05 (17)	C12—C13—O7—C19	−178.35 (16)
C4—C5—C6—C1	−1.7 (2)	C13—C14—C15—C10	0.5 (3)
C4—C5—C6—O1	177.63 (11)	C14—C13—O7—C19	2.6 (2)
C4—C5—C7—C8	179.28 (13)	C15—C10—C11—C12	−1.3 (2)
C4—C5—C7—O5	−1.3 (2)	O1—C9—C10—C11	32.96 (17)
C5—C6—O1—C9	4.11 (18)	O1—C9—C10—C15	−146.90 (14)
C5—C7—C8—C9	2.3 (2)	O2—C2—C3—C4	178.76 (12)
C5—C7—C8—O6	175.71 (12)	O2—C2—C3—O3	−4.78 (19)
C6—C1—C2—C3	1.4 (2)	O3—C3—C4—C5	−176.13 (12)
C6—C1—C2—O2	−179.15 (13)	O3—C3—C4—O4	4.0 (2)
C6—C5—C7—C8	−1.2 (2)	O4—C4—C5—C6	−178.79 (13)
C6—C5—C7—O5	178.25 (13)	O4—C4—C5—C7	0.8 (2)
C7—C5—C6—C1	178.74 (12)	O5—C7—C8—C9	−177.12 (14)
C7—C5—C6—O1	−2.0 (2)	O5—C7—C8—O6	−3.7 (2)
C7—C8—C9—C10	175.13 (13)	O6—C8—C9—C10	1.6 (2)
C7—C8—C9—O1	−0.3 (2)	O6—C8—C9—O1	−173.81 (12)
C7—C8—O6—C18	67.14 (19)	O7—C13—C14—C15	177.69 (15)

### Hydrogen-bond geometry (Å, °)

**Table:**

*D*—H···*A*	*D*—H	H···*A*	*D*···*A*	*D*—H···*A*
O4—H4···O5	0.82	1.89	2.6157 (16)	147.
